# Health-related quality of life after treatment for bladder cancer in England

**DOI:** 10.1038/s41416-018-0084-z

**Published:** 2018-05-14

**Authors:** Samantha J Mason, Amy Downing, Penny Wright, Luke Hounsome, Sarah E Bottomley, Jessica Corner, Mike Richards, James W Catto, Adam W Glaser

**Affiliations:** 1 Leeds Institute of Cancer and Pathology, University of Leeds, Level 11, Worsley Building, Clarendon Way, Leeds, LS2 9NL UK; 2 Leeds Institute for Data Analytics, University of Leeds, Level 11, Worsley Building, Clarendon Way, Leeds, LS2 9NL UK; 3 Public Health England, 2 Rivergate, Temple Quay, Bristol, BS1 6EH UK; 4 Academic Urology Unit, University of Sheffield, The Medical School, Beech Hill Road, Sheffield, S10 2RX UK; 5 Executive Office, University of Nottingham, Trent Building, University Park, Nottingham, NG7 2RD UK; 6 London, UK

**Keywords:** Bladder cancer, Cancer epidemiology

## Abstract

**Background:**

Little is known about quality of life after bladder cancer treatment. This common cancer is managed using treatments that can affect urinary, sexual and bowel function.

**Methods:**

To understand quality of life and inform future care, the Department of Health (England) surveyed adults surviving bladder cancer 1–5 years after diagnosis. Questions related to disease status, co-existing conditions, generic health (EQ-5D), cancer-generic (Social Difficulties Inventory) and cancer-specific outcomes (Functional Assessment of Cancer Therapy—Bladder).

**Results:**

In total, 673 (54%) patients responded; including 500 (74%) men and 539 (80%) with co-existing conditions. Most respondents received endoscopic treatment (60%), while 92 (14%) and 99 (15%) received radical cystectomy or radiotherapy, respectively. Questionnaire completion rates varied (51–97%). Treatment groups reported ≥1 problem using EQ-5D generic domains (59–74%). Usual activities was the most common concern. Urinary frequency was common after endoscopy (34–37%) and radiotherapy (44–50%). Certain populations were more likely to report generic, cancer-generic and cancer-specific problems; notably those with co-existing long-term conditions and those treated with radiotherapy.

**Conclusion:**

The study demonstrates the importance of assessing patient-reported outcomes in this population. There is a need for larger, more in-depth studies to fully understand the challenges patients with bladder cancer face.

## Introduction

Bladder cancer (BC) is the 9^th^ most common cancer in the United Kingdom and one of the most expensive malignancies to manage.^1,2^ The disease is best stratified according to the presence of muscle invasion and cellular differentiation. Most BCs are non-muscle invasive (NMIBC) and have an excellent long-term prognosis.^3^ NMIBC tumours are managed by endoscopic resection, intravesical chemotherapy and long-term surveillance.^4^ Following initial treatment, many patients develop local recurrence, requiring further treatments.^5^ Around 1/3 of tumours are muscle invasive BCs (MIBCs), requiring radical treatment if cure is to be obtained. Radical cystectomy (RC) or radiotherapy includes treatment of adjacent viscera with regional lymph nodes, and often includes systemic chemotherapy. The nature and toxicity of treatments and surveillance for BC can vary between patients, between each option and over time. There is evidence that treatment for MIBC can impact upon urinary function,^6^ bowel function,^7^ sexual function,^8,9^ and affects body image,^10,11^ which can lead to anxiety and depression.^7^ However, there is less evidence regarding the consequences of treatment for NMIBC and the impact on patients' Health-Related Quality of Life (HRQL).^12,13^

The importance of large scale, population-level Patient-Reported Outcome Measures (PROMS) in improving healthcare design, patient experience and directing care is becoming recognised.^14,15^ PROMS can be used to ascertain a more comprehensive understanding of the quality of survival, alongside the impact and relevance of health care provision, and as a surrogate measure within clinical trials. Previous research in the USA used a linkage database to identify BC patients and looked at results of 620 surveys completed before diagnosis and 856 completed after by patients ≥65 years old.^16^ European PROMs work included 823 German patients of all ages and stages of BC.^13^ These cross-sectional studies used generic PROMs or generic cancer PROMs.

To date, no large-scale surveys of BC patients have been conducted in the United Kindgom. As such, in 2013 the Department of Health (DH) England designed and administered a pilot survey of patients 1–5 years following their initial treatment for BC. Here we report the results of the pilot survey, which was conducted to identify a methodology to define the HRQL of individuals in the years following their treatment and to identify potential factors associated with poor outcomes.

## Methods

### Survey design

The DH methodology has been described previously for cohorts diagnosed with non-Hodgkin’s lymphoma, breast, colorectal and prostate cancer.^17^ Individuals aged 16 or older surviving 1–5 years after a diagnosis of BC were identified via the Eastern Cancer Registration and Intelligence Centre (now part of National Cancer Registration and Analysis Service, NCRAS).^18^ The sample size was chosen to match similar studies performed by the DH in other cancer sites.^17^

Identified participants were mailed a questionnaire, with a covering letter from their treating Cancer Centre. Consent to participate was implied through return of completed questionnaires. Individuals who did not want to participate were asked to return their questionnaire uncompleted or to discard the survey. Two reminders were sent to non-responders. A Freephone helpline for patients was provided, which supported completion of the survey. Permission to approach patients without informed consent was given by the Health Research Authority (ref ECC 5-02(FT7)/2012).

### Survey content

Survey content included questions about treatment, disease status, generic HRQL (EQ-5D-5L) and BC specific outcomes (Functional Assessment of Cancer Therapy—Bladder (FACT-Bl)), social problems (Social Difficulties Inventory (SDI-21)), health and well-being in the past month, experience of care and presence of other long-term conditions (LTCs) (Supplementary File 1).

The EQ-5D-5L records problems on five domains (mobility, self-care, usual activities, pain/discomfort and anxiety/depression).^19,20^ There are five response options ranging from no problems to extreme problems. Respondents are asked to complete the response options based on how they are feeling that day.

The SDI-21 is a 21-item questionnaire, developed to assess everyday problems experienced by cancer patients.^21^ Questions are answered on a scale of 0 (no difficulty) to 3 (very much), with respect to the past month. Sixteen of the items form three subscales: Everyday Living, Money Matters and Self and Others. These scales form a measure of social distress (SD-16), with scores ranging from 0 to 44.^22^ The SDI-21 also comprises five single items.

FACT-Bl consists of the 27-item FACT-General (FACT-G) questionnaire^23^ and 13 additional items. FACT-G covers four areas; Physical well-being, Social/family well-being, Emotional well-being and Functional well-being. The 13 additional items relate to urinary issues, bowel issues, appetite and weight, sexual items, body image, a question asking if the respondent has an ostomy appliance and two questions about ostomy appliances. All items ask about the last 7 days.

These surveys were chosen for inclusion as EQ-5D-5L, SDI-21 and Functional Assessment of Chronic Illness Therapy (FACIT) modules have been used in similar studies performed by the DH in other cancer sites.^17,14^ Cognitive testing of all questionnaires was performed with a group of volunteer patients and expert panel review (clinicians/methodologists). In the final version of the survey, the team designing the survey removed the 'somewhat' response option from FACT-Bl; changing the questionnaire from five responses to four.^18^

### Data handling

All variables were derived from the survey data. Participants were asked if they had any other LTCs at the time of completing the questionnaire and to tick all conditions that they had from a list widely used in English DH surveys. This variable was categorised into none, 1, 2 or ≥3 LTCs. Information on self-reported disease status (in remission, treated but still present, not treated, recurrence, and not certain) and treatments (endoscopic/telescopic surgery with or without chemotherapy directly into the bladder, RC, chemotherapy, and radiotherapy) was taken from the questionnaire. Age was grouped into <55 years, 55–64 years, 65–74 years, 75–85 years and ≥85 years.

EQ-5D-5L responses were split into people who reported at least one problem (of any severity) on any domain and people who reported having no problems on any domain. Individual domains were categorised in this way. A validated cutoff score of ≥10 on the SD-16 scale indicates a high level of social difficulties that requires follow-up by health or social care staff.^24^ This was used in our analysis as a cutoff point (socially distressed v not socially distressed). Estimated cutoff points of 5 for the Everyday Living subscale, 2 for the Money Matters subscale and 3 for the Self and Others subscale were used in this study, as per previous research.^25^ The five single items of the SDI-21 are scored individually.^22^ As the 'somewhat' option was removed from the questionnaire, FACT-Bl scores could not be calculated as per normal practice and thus cancer-specific questions from FACT-Bl were examined separately. FACT-Bl responses were grouped into those who responded 'not at all' or 'a little' and those who responded 'quite a bit' and 'very much'. Outcomes pertaining to well-being, urinary items, sexual items and body image are presented here.

### Statistical analysis

Descriptive statistics were used to report respondent characteristics, EQ-5D-5L responses, SD-16 scores, SDI-21 subscale scores and FACT-Bl responses. Outcomes were analysed in relation to age, sex, other comorbidities and type of treatment using *χ*^2^ tests. Statistical significance was set at the 1% level to minimise the chances of false-positive associations. Analyses were performed using Stata version 15 (Stata, College Station, TX).

## Results

### Survey population

**Fig. 1:**
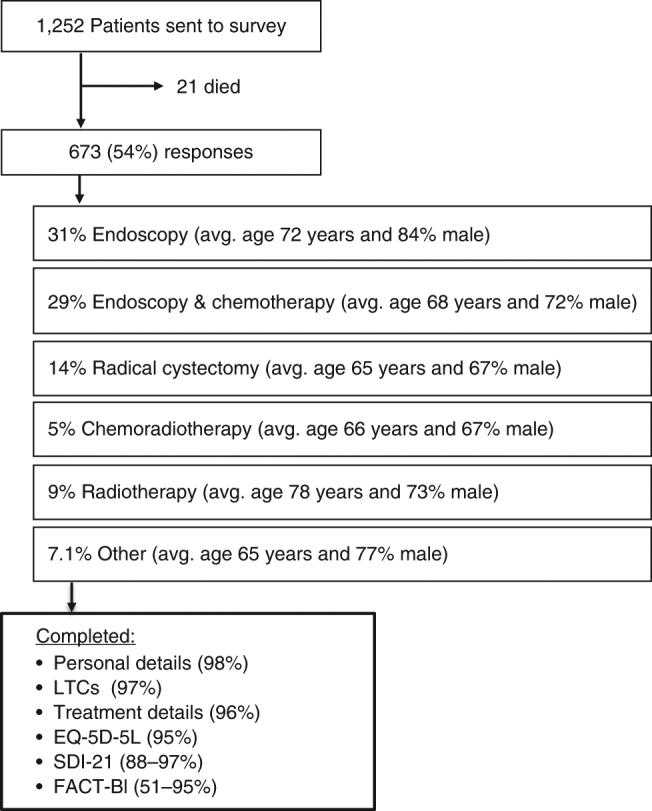
Design and response rates within this survey

In total, 1252 BC patients were randomly identified and sent a questionnaire (Fig. 1). Of these, 21 (2%) died during the survey period, leaving 1231 eligible patients. Questionnaires were returned by 673 people (54% response rate), including 500 (74%) men and 162 (24%) women (Table 1). Most respondents were white (93%) and were in remission from BC (65%). Co-existing LTCs were common (80% reported ≥1 LTC and 29% reported ≥3). The most common treatment was endoscopy/telescopy (31%). Radical treatment was reported by 28% of respondents: of which 14% had undergone RC, 9% had received external beam radiotherapy and 5% had radiotherapy with intravenous chemotherapy. Other treatment combinations were given to <2% of respondents and therefore excluded from analysis. A stoma was present in 16% of respondents. Of the radical treatments, patients ≥85 years were more likely to be treated with radiotherapy (31%) (Supplementary Table 1).

**Table 1:** Demographics of survey respondents

Demographic	No. of respondents	%
*Age, years*		
<55	53	7.9
55–64	137	20.4
65–74	247	36.7
75–84	184	27.3
≥85	35	5.2
No response	17	2.5
*Sex*		
Male	500	74.3
Female	162	24.1
Not known	11	1.6
*Race*		
White	667	99.1
Non white	6	0.9
*No. of long-term conditions (LTCs)*		
None	111	16.5
1	205	30.4
2	141	21.0
≥3	193	28.7
Not reported	23	3.4
*Disease status*		
Remission	434	64.5
Treated but cancer still present	42	6.2
No treatment	2	0.3
Recurrence	30	4.5
Not certain	85	12.6
Not reported	80	11.9
*Treatment*		
Endoscopy/telescopy	207	30.8
Endoscopy/telescopy with chemotherapy directly into the bladder	198	29.4
Radical cystectomy	92	13.7
Radiotherapy and Intravenous chemotherapy	36	5.3
Radiotherapy	63	9.4
Other	48	7.1
Not reported	29	4.3
*Stoma status*		
Stoma	108	16.0
No stoma	437	65.0
Not reported	128	19.0

Respondent and non-respondent characteristics were compared, using data from NCRAS (Supplementary Table 2). Individuals older than 85 years (RR, 39%) were less likely to participate.

### Data quality

Most patients answered questions relating to sex, LTCs and treatment (<5% missing responses). Of all the PROMs, FACT-Bl had the largest variety of completion rates for items and scales; with missing responses ranging from 5 to 49% (Supplementary Table 3).

### Generic HRQL

Overall, 65% of respondents reported ≥1 problem on any EQ-5D-5L domain (Table 2). The percentage of respondents from treatment groups reporting ≥1 problem on any EQ-5D-5L domain ranged from 59% for endoscopy/telescopy and intravesical chemotherapy to 74% for radiotherapy. Problems with usual activities were most commonly reported (43%). Respondents treated with radiotherapy reported more problems with mobility, self-care and usual activities compared to respondents who received other treatments. Respondents with endoscopy/telescopy were more likely to report problems with mobility than respondents treated with endoscopy/telescopy and intravesical chemotherapy (47% compared to 26%, *p* < 0.01).

**Table 2:** EQ-5D-5L number of generic health problems and domain responses by demographic

Demographic	Problems on any EQ5D domain					Mobility					Self-care				
	No problems		Problems		*p*-value	No problems		Problems		*p*-value	No problems		Problems		*p*-value
	N	%	N	%		N	%	N	%		N	%	N	%	
*Age, years*					*χ*^2^ = 5.7, *p* = 0.226					*χ*^2^ = 41.8, *p* < 0.01					*χ*^2^ = 16.4, *p* < 0.01
<55	21	41.2	30	58.8		40	76.9	12	23.1		45	88.2	6	11.8	
55–64	43	32.6	89	67.4		101	74.3	35	25.7		119	87.5	17	12.5	
65–74	92	39.5	141	60.5		164	68.3	76	31.7		210	87.1	31	12.9	
75–84	54	30.9	121	69.1		88	49.2	91	50.8		141	77.0	42	23.0	
≥85	9	26.5	25	73.5		12	34.3	23	65.7		23	67.7	11	32.3	
*Sex*					*χ*^2^ = 0.2, *p* = 0.651					*χ*^2^ = 0.3, *p* = 0.584					*χ*^2^ = 0, *p* = 0.911
Male	170	35.6	308	64.4		310	63.7	177	36.3		410	83.5	81	16.5	
Female	51	33.6	101	66.4		98	61.2	62	38.8		133	83.1	27	16.9	
*No. of long-term conditions*					*χ*^2^ = 48.9, *p* < 0.01					*χ*^2^ = 115.9, *p* < 0.01					*χ*^2^ = 81.9, *p* < 0.01
None	57	53.3	50	46.7		94	86.2	15	13.8		107	98.2	2	1.8	
1	84	43.5	109	56.5		155	78.3	43	21.7		181	91.0	18	9.0	
2	41	30.1	95	69.9		84	60.4	55	39.6		120	86.3	19	13.7	
≥3	31	17.1	150	82.9		63	33.3	126	66.7		120	62.8	71	37.2	
*Disease status*					*χ*^2^ = 17.4, *p* = 0.01					*χ*^2^ = 13, *p* < 0.01					*χ*^2^ = 11.5, *p* < 0.01
Remission	170	40.4	251	59.6		288	66.8	143	33.2		371	85.9	61	14.1	
Treated but cancer still present	9	22.5	31	77.5		21	51.2	20	48.8		29	70.7	12	29.3	
Recurrence	8	26.7	22	73.3		20	66.7	10	33.3		25	83.3	5	16.7	
Not certain	15	19.2	63	80.8		39	48.1	42	51.9		60	74.1	21	25.9	
*Treatment*					*χ*^2^ = 6.6, *p* = 0.156					*χ*^2^ = 31.7, *p* < 0.01					*χ*^2^ = 20.5, *p* < 0.01
Endoscopy/telescopy	68	34.5	129	65.5		108	53.5	94	46.5		166	81.0	39	19.0	
Endoscopy/telescopy with chemotherapy directly into the bladder	80	41.5	113	58.5		145	74.0	51	26.0		177	89.9	20	10.1	
Radical cystectomy	27	30.7	61	69.3		62	68.9	28	31.1		74	82.2	16	17.8	
Radiotherapy and Intravenous chemotherapy	10	30.3	23	69.7		20	57.1	15	42.9		31	88.6	4	11.4	
Radiotherapy	16	26.2	45	73.8		26	41.3	37	58.7		41	66.1	21	33.9	
*Stoma status*					*χ*^2^ = 1.7, *p* = 0.189					*χ*^2^ = 0.2, *p* = 0.622					*χ*^2^ = 0.7, *p* = 0.400
Stoma	31	29.5	74	70.5		70	66.0	36	34.0		86	81.1	20	18.9	
No stoma	152	36.4	266	63.6		271	63.5	156	36.5		365	84.5	67	15.5	

Demographic	Usual activities					Pain/Discomfort					Anxiety/Depression				
	No problems		Problems		*p*-value	No problems		Problems		*p*-value	No problems		Problems		*p*-value
	N	%	N	%		N	%	N	%		N	%	N	%	
*Age, years*					*χ*^2^ = 21, *p* < 0.01					*χ*^2^ = 7.7, *p* = 0.103					*χ*^2^ = 13.3, *p* = 0.01
<55	35	67.3	17	32.7		31	59.6	21	40.4		26	50.0	26	50.0	
55–64	84	62.7	50	37.3		74	54.4	62	45.6		76	56.3	59	43.7	
65–74	151	63.2	88	36.8		161	67.9	76	32.1		165	69.0	74	31.0	
75–84	87	47.5	96	52.5		118	64.5	65	35.5		125	69.4	55	30.6	
≥85	12	35.3	22	64.7		20	57.1	15	42.9		20	58.8	14	41.2	
*Sex*					*χ*^2^ = 3.2, *p* = 0.073					*χ*^2^ = 1.9, *p* = 0.169					*χ*^2^ = 0, *p* = 0.876
Male	291	59.4	199	40.6		300	61.2	190	38.8		317	64.6	174	35.4	
Female	81	51.3	77	48.7		107	67.3	52	32.7		99	63.9	56	36.1	
*No. of long-term conditions*					*χ*^2^ = 74.4, *p* < 0.01					*χ*^2^ = 41.8, *p* < 0.01					*χ*^2^ = 10.8, *p* = 0.013
None	87	79.1	23	20.9		87	79.8	22	20.2		79	71.8	31	28.2	
1	133	67.2	65	32.8		140	69.7	61	30.3		132	67.0	65	33.0	
2	78	56.1	61	43.9		82	59.9	55	40.1		91	65.9	47	34.1	
≥3	62	33.0	126	67.0		86	45.5	103	54.5		103	54.8	85	45.2	
*Disease status*					*χ*^2^ = 19.6, *p* < 0.01					*χ*^2^ = 18, *p* < 0.01					*χ*^2^ = 16.9, *p* < 0.01
Remission	271	63.3	157	36.7		292	67.9	138	32.1		301	70.2	128	29.8	
Treated but cancer still present	20	48.8	21	51.2		21	52.5	19	47.5		19	47.5	21	52.5	
Recurrence	16	53.3	14	46.7		16	53.3	14	46.7		15	50.0	15	50.0	
Not certain	31	38.3	50	61.7		37	45.7	44	54.3		44	55.0	36	45.0	
*Treatment*					*χ*^2^ = 19.2, *p* < 0.01					*χ*^2^ = 14.3, *p* < 0.01					*χ*^2^ = 2.1, *p* = 0.719
Endoscopy/telescopy	123	60.0	82	40.0		144	70.6	60	29.4		132	65.4	70	34.6	
Endoscopy/telescopy with chemotherapy directly into the bladder	131	66.8	65	33.2		130	66.7	65	33.3		129	65.5	68	34.5	
Radical cystectomy	41	46.1	48	53.9		51	56.0	40	44.0		53	59.6	36	40.4	
Radiotherapy and Intravenous chemotherapy	17	50.0	17	50.0		16	47.1	18	52.9		20	57.1	15	42.9	
Radiotherapy	26	41.9	36	58.1		33	53.2	29	46.8		41	67.2	20	32.8	
*Stoma status*					*χ*^2^ = 6, *p* = 0.014					*χ*^2^ = 7.2, *p* < 0.01					*χ*^2^ = 0.1, *p* = 0.807
Stoma	51	47.7	56	52.3		54	50.5	53	49.5		67	62.6	40	37.4	
No stoma	260	60.7	168	39.3		277	64.6	152	35.4		276	63.9	156	36.1	

Respondents aged ≥85 years were most likely to report some problems with mobility, self-care and usual activities. Respondents <55 years old were significantly more likely to report problems with anxiety/depression, with half of this group reporting some problems, compared to between 31–44% of other age groups (*p* = 0.01). Those with ≥3 LTCs reported significantly more problems on all EQ-5D-5L domains bar one (anxiety/depression).

### Social difficulties

#### SD-16

Overall, 15% of respondents were classed as socially distressed (score ≥10, Table 3). No differences were observed by sex or age group. The respondents most likely to report significantly high social distress were those treated with radiotherapy and respondents with ≥3 LTCs; with more than a quarter of respondents from these groups meeting the criteria. Respondents with a stoma were twice as likely to be socially distressed compared to respondents without a stoma.

**Table 3:** Social Difficulties Inventory: SD-16 and SDI-21 subscale results by demographic

Demographic	Social Distress				*p*-value	Everyday Living				*p*-value	Money Matters				*p*-value	Self and Others				*p*-value
	SD		No SD			Difficulty		No difficulty			Difficulty		No difficulty			Difficulty		No difficulty		
	N	%	N	%		N	%	N	%		N	%	N	%		N	%	N	%	
*Age, years*					*χ*^2^ = 7.8, *p* = 0.098					*χ*^2^ = 8.6, *p* = 0.071					*χ*^2^ = 57.9, *p* < 0.01					*χ*^2^ = 24.9, *p* < 0.01
<55	14	26.4	39	73.6		13	24.5	40	75.5		22	41.5	31	58.5		18	34.0	35	66.0	
55–64	23	17.3	110	82.7		22	16.3	113	83.7		31	23.3	102	76.7		34	25.0	102	75.0	
65–74	29	12.0	212	88.0		44	18.1	199	81.9		23	9.5	218	90.5		29	11.9	214	88.1	
75–84	23	13.8	144	86.2		43	24.0	136	76.0		11	6.5	157	93.5		21	11.9	156	88.1	
≥85	5	16.1	26	83.9		12	35.3	22	64.7		1	3.0	32	97.0		6	18.7	26	81.3	
*Sex*					*χ*^2^ = 0.2, *p* = 0.688					*χ*^2^ = 4.7, *p* = 0.031					*χ*^2^ = 3.4, *p* = 0.065					*χ*^2^ = 0.5, *p* = 0.484
Male	71	14.8	410	85.2		93	18.9	400	81.1		74	15.3	410	84.7		80	16.3	411	83.7	
Female	24	16.1	125	83.9		42	26.9	114	73.1		14	9.3	136	90.7		29	18.7	126	81.3	
*No. of long-term conditions*					*χ*^2^ = 26, *p* < 0.01					*χ*^2^ = 64.7, *p* < 0.01					*χ*^2^ = 4.4, *p* = 0.221					*χ*^2^ = 8.2, *p* = 0.041
None	6	5.7	100	94.3		9	8.3	99	91.7		11	10.4	95	89.6		12	11.1	96	88.9	
1	23	11.9	170	88.1		25	12.3	178	87.7		25	12.8	170	87.2		34	17.1	165	82.9	
2	18	13.4	116	86.6		25	18.1	113	81.9		17	12.7	117	87.3		19	13.8	119	86.2	
≥3	48	26.1	136	73.9		77	41.0	111	59.0		34	18.3	152	81.7		43	22.9	145	77.1	
*Disease status*					*χ*^2^ = 18.8, *p* < 0.01					*χ*^2^ = 14.9, *p* < 0.01					*χ*^2^ = 5.6, *p* = 0.134					*χ*^2^ = 15.2, *p* < 0.01
Remission	43	10.3	376	89.7		71	16.5	359	83.5		48	11.4	373	88.6		53	12.4	373	87.6	
Treated but cancer still present	10	24.4	31	75.6		12	29.3	29	70.7		4	9.8	37	90.2		7	17.1	34	82.9	
Recurrence	6	20.7	23	79.3		6	20.7	23	79.3		6	20.7	23	79.3		9	30.0	21	70.0	
Not certain	20	26.0	57	74.0		27	33.7	53	66.3		15	19.2	63	80.8		21	26.6	58	73.4	
*Treatment*					*χ*^2^ = 22.1, *p* < 0.01					*χ*^2^ = 27.7, *p* < 0.01					*χ*^2^ = 7.1, *p* = 0.131					*χ*^2^ = 18.1, *p* < 0.01
Endoscopy/telescopy	23	11.6	176	88.4		47	23.1	156	76.9		23	11.6	176	88.4		26	12.9	176	87.1	
Endoscopy/telescopy with chemotherapy directly into the bladder	16	8.5	173	91.5		21	10.8	174	89.2		22	11.6	167	88.4		22	11.2	174	88.8	
Radical cystectomy	21	23.6	68	76.4		23	25.3	68	74.7		17	19.1	72	80.9		25	27.8	65	72.2	
Radiotherapy and Intravenous chemotherapy	7	20.0	28	80.0		7	20.0	28	80.0		7	20.0	28	80.0		7	20.0	28	80.0	
Radiotherapy	16	28.1	41	71.9		24	40.7	35	59.3		4	6.9	54	93.1		15	25.4	44	74.6	
*Stoma status*					*χ*^2^ = 9.4, *p* < 0.01					*χ*^2^ = 1.1, *p* = 0.293					*χ*^2^ = 3.3, *p* = 0.068					*χ*^2^ = 6, *p* = 0.014
Stoma	24	22.6	82	77.4		25	23.1	83	76.9		20	18.9	86	81.1		26	24.1	82	75.9	
No stoma	48	11.2	379	88.8		81	18.7	353	81.3		52	12.1	377	87.9		62	14.3	371	85.7	

#### SDI-21 subscales

Difficulties with Everyday Living (score ≥5) were reported by 21% of respondents (Table 3). Respondents treated with radiotherapy and those who had ≥3 LTCs reported a higher level of difficulty with Everyday Living (both 41%). Comparatively fewer patients receiving other treatments reported difficulties (≤25%). When comparing patients who did not have radical treatments, significantly more respondents with endoscopy/telescopy reported difficulties with Everyday Living than respondents treated with endoscopy/telescopy and intravesical chemotherapy (23% compared to 11%, *p* < 0.01). Difficulties with Everyday Living did not vary by sex, age group or stoma status.

Difficulties with Money Matters (score ≥2) were reported by 14% of respondents. This difficulty was significantly more likely to be reported by respondents who were <55 years of age (42% compared to between 3 and 23% of other age groups, *p* < 0.01). Differences were not found for treatment type, disease status, stoma status, LTCs or sex (Table 3).

Difficulties with Self and Others (score ≥3) were reported by 17% of respondents. Reporting of significant difficulties with Self and Others was high in respondents <55 years of age, where more than a third (34%) reported difficulties (Table 3).

#### SDI-21 single items

The most commonly reported difficulty was with travelling or plans to take a holiday; reported by 33% of respondents. Respondents with a stoma were significantly more likely to report 'quite a bit' or 'very much' difficulty with this item than those without a stoma (29% compared to 15%, *p* < 0.01).

Difficulties with sexual matters were reported 'quite a bit' or 'very much' by 15% of respondents. This difficulty was significantly more likely to impact on men, 17% of whom reported 'quite a bit' or 'very much' difficulty compared to 5% of females (*p* < 0.01).

### Cancer-specific HRQL

#### Physical well-being

Overall, 25% of the cohort responded that they experienced a lack of energy 'quite a bit' or 'very much', but this was higher in respondents treated with radiotherapy (43%) (Table 4).

**Table 4:** Cancer-specific patient-reported outcomes by treatment group

	Endoscopy/telescopy				Endoscopy/telescopy with chemotherapy directly into the bladder				Radical cystectomy				Radiotherapy and Intravenous chemotherapy				Radiotherapy				*p*-value
	Not at all/somewhat		Quite a bit/very much		Not at all/somewhat		Quite a bit/very much		Not at all/somewhat		Quite a bit/very much		Not at all/somewhat		Quite a bit/very much		Not at all/somewhat		Quite a bit/very much		
	N	%	N	%	N	%	N	%	N	%	N	%	N	%	N	%	N	%	N	%	
*Physical well-being*																					
I have a lack of energy	150	76.9	45	23.1	155	83.3	31	16.7	68	78.2	19	21.8	25	73.5	9	26.5	34	56.7	26	43.3	*χ*^2^ = 18.4, *p* < 0.01
Because of my physical condition, I have trouble meeting the needs of my family	162	90.5	17	9.5	173	96.1	7	3.9	77	88.5	10	11.5	29	87.9	4	12.1	39	78.0	11	22.0	*χ*^2^ = 16.7, *p* < 0.01
I have pain	165	91.2	16	8.8	165	91.7	15	8.3	78	90.7	8	9.3	30	90.9	3	9.1	45	81.8	10	18.2	*χ*^2^ = 5.1, *p* = 0.280
*Social/family well-being*																					
I feel close to my friends	72	42.9	96	57.1	51	30.0	119	70.0	23	27.7	60	72.3	16	47.1	18	52.9	23	45.1	28	54.9	*χ*^2^ = 12, *p* = 0.017
My family has accepted my illness	36	20.7	138	79.3	21	11.9	156	88.1	6	7.1	79	92.9	5	14.7	29	85.3	6	10.9	49	89.1	*χ*^2^ = 10.9, *p* = 0.027
I am satisfied with my sex life	60	60.6	39	39.4	68	59.7	46	40.3	44	88.0	6	12.0	11	64.7	6	35.3	17	73.9	6	26.1	*χ*^2^ = 14.8, *p* < 0.01
*Emotional well-being*																					
I feel sad	100	88.5	13	11.5	121	91.0	12	9.0	57	85.1	10	14.9	18	85.7	3	14.3	29	87.9	4	12.1	*χ*^2^ = 1.8, *p* = 0.781
I am satisfied with how I am coping with my illness	44	34.9	82	65.1	30	21.6	109	78.4	15	20.5	58	79.5	5	21.7	18	78.3	11	29.7	26	70.3	*χ*^2^ = 8.1, *p* = 0.087
I feel nervous	106	86.2	17	13.8	124	90.5	13	9.5	64	90.1	7	9.9	21	95.5	1	4.5	32	97.0	1	3.0	*χ*^2^ = 4.5, *p* = 0.343
I worry about dying	112	89.6	13	10.4	129	92.1	11	7.9	63	90.0	7	10.0	20	90.9	2	9.1	32	94.1	2	5.9	*χ*^2^ = 1, *p* = 0.907
I worry that my condition will get worse	166	86.9	25	13.1	163	87.2	24	12.8	75	86.2	12	13.8	21	63.6	12	36.4	47	83.9	9	16.1	*χ*^2^ = 13.3, *p* = 0.01
*Functional well-being*																					
I am able to work (include work from home)	63	35.8	113	64.2	47	26.5	130	73.5	31	36.5	54	63.5	11	36.7	19	63.3	28	58.3	20	41.7	*χ*^2^ = 17.2, *p* < 0.01
My work (include work at home) is fulfilling	56	33.9	109	66.1	48	28.6	120	71.4	31	38.3	50	61.7	8	28.6	20	71.4	26	57.8	19	42.2	*χ*^2^ = 14.3, *p* < 0.01
I am able to enjoy life	46	24.5	142	75.5	32	16.8	159	83.2	22	24.7	67	75.3	2	6.1	31	93.9	17	28.8	42	71.2	*χ*^2^ = 10.7, *p* = 0.03
I am sleeping well	66	34.2	127	65.8	52	28.0	134	72.0	29	32.2	61	67.8	12	35.3	22	64.7	18	31.0	40	69.0	*χ*^2^ = 2, *p* = 0.739
I am enjoying the things I usually do for fun	53	28.5	133	71.5	37	20.0	148	80.0	25	29.1	61	70.9	11	33.3	22	66.7	21	38.9	33	61.1	*χ*^2^ = 9.5, *p* = 0.049
I am content with the quality of my life right now	52	27.7	136	72.3	34	17.8	157	82.2	28	32.2	59	67.8	10	30.3	23	69.7	16	28.6	40	71.4	*χ*^2^ = 9.2, *p* = 0.057
*Bladder cancer-specific items*																					
I have control of my bowels	42	21.6	152	78.4	25	13.0	167	87.0	20	23.0	67	77.0	5	15.1	28	84.9	14	24.1	44	75.9	*χ*^2^ = 7.6, *p* = 0.107
I urinate more frequently than usual	127	65.5	67	34.5	120	62.8	71	37.2	NA	NA	NA	NA	17	50.0	17	50.0	29	55.8	23	44.2	*χ*^2^ = 4, *p* = 0.264
I have a good appetite	43	21.7	155	78.3	25	13.0	167	87.0	19	22.1	67	77.9	4	11.4	31	88.6	14	23.7	45	76.3	*χ*^2^ = 8.2, *p* = 0.085
It burns when I urinate	179	93.7	12	6.3	165	91.2	16	8.8	NA	NA	NA	NA	29	85.3	4	14.7	53	93.0	4	7.0	*χ*^2^ = 3.1, *p* = 0.377
I am interested in sex	110	65.9	57	34.1	106	60.2	70	39.8	55	71.4	22	28.6	23	71.9	9	28.1	39	81.3	9	18.7	*χ*^2^ = 9.1, *p* = 0.059
(For men only) I am able to have and maintain an erection	91	66.4	46	33.6	76	59.8	51	40.2	52	94.5	3	5.5	17	89.5	2	10.5	34	89.5	4	10.5	NA

Pain was reported 'quite a bit' or 'very much' by 10% of respondents and was higher in respondents with ≥3 LTCs (19%) (Table 5).

**Table 5:** Cancer-specific patient-reported outcomes by number of long-term conditions (LTCs)

	No LTC				1 LTC				2 LTCs				≥3 LTCs				*p*-value
	Not at all/somewhat		Quite a bit/very much		Not at all/somewhat		Quite a bit/very much		Not at all/somewhat		Quite a bit/very much		Not at all/somewhat		Quite a bit/very much		
	N	%	N	%	N	%	N	%	N	%	N	%	N	%	N	%	
*Physical well-being*																	
I have a lack of energy	97	91.5	9	8.5	170	85.9	28	14.1	98	76.0	31	24.0	101	55.5	81	44.5	*χ*^2^ = 66, *p* < 0.01
Because of my physical condition, I have trouble meeting the needs of my family	104	98.1	2	1.9	170	91.9	15	8.1	116	94.3	7	5.7	132	80.0	33	20.0	*χ*^2^ = 29.8, *p* < 0.01
I have pain	101	97.1	3	2.9	175	94.6	10	5.4	109	87.2	16	12.8	138	81.2	32	18.8	*χ*^2^ = 24.9, *p* < 0.01
*Social/family well-being*																	
I feel close to my friends	26	27.4	69	72.6	64	35.4	117	64.6	46	39.3	71	60.7	66	41.0	95	59.0	*χ*^2^ = 5.3, *p* = 0.150
My family has accepted my illness	14	13.6	89	86.4	25	13.8	156	86.2	20	16.3	103	83.7	18	10.8	148	89.2	*χ*^2^ = 1.8, *p* = 0.609
I am satisfied with my sex life	39	56.5	30	43.5	71	61.7	44	38.3	48	75.0	16	25.0	67	76.1	21	23.9	*χ*^2^ = 10, *p* = 0.018
*Emotional well-being*																	
I feel sad	70	94.6	4	5.4	128	94.1	8	5.9	74	88.1	10	11.9	86	76.1	27	23.9	*χ*^2^ = 22.9, *p* < 0.01
I am satisfied with how I am coping with my illness	16	20.8	61	79.2	31	21.8	111	78.2	22	23.7	71	76.3	47	36.2	83	63.8	*χ*^2^ = 9.5, *p* = 0.023
I feel nervous	73	97.3	2	2.7	133	94.3	8	5.7	84	93.3	6	6.7	95	79.2	25	20.8	*χ*^2^ = 24.9, *p* < 0.01
I worry about dying	69	90.8	7	9.2	132	93.6	9	6.4	85	94.4	5	5.6	104	83.9	20	16.1	*χ*^2^ = 9.6, *p* = 0.023
I worry that my condition will get worse	91	85.8	15	14.2	166	86.5	26	13.5	113	87.6	16	12.4	145	81.0	34	19.0	*χ*^2^ = 3.3, *p* = 0.348
*Functional well-being*																	
I am able to work (include work at home)	18	18.0	82	82.0	38	21.3	140	78.7	53	42.7	71	57.3	87	53.7	75	46.3	*χ*^2^ = 55.6, *p* < 0.01
My work (include work at home) is fulfilling	20	21.5	73	78.5	44	26.0	125	74.0	43	36.8	74	63.2	77	51.3	73	48.7	*χ*^2^ = 31.2, *p* < 0.01
I am able to enjoy life	16	15.0	91	85.0	29	14.7	168	85.3	25	19.4	104	80.6	60	33.7	118	66.3	*χ*^2^ = 24.3, *p* < 0.01
I am sleeping well	23	21.9	82	78.1	52	26.8	142	73.2	44	33.1	89	66.9	75	41.9	104	58.1	*χ*^2^ = 15.5, *p* < 0.01
I am enjoying the things I usually do for fun	16	15.7	86	84.3	31	16.2	160	83.8	34	27.2	91	72.8	79	45.4	95	54.6	*χ*^2^ = 47.7, *p* < 0.01
I am content with the quality of my life right now	22	20.4	86	79.6	28	14.6	164	85.4	38	29.9	89	70.1	67	37.4	112	62.6	*χ*^2^ = 28.2, *p* < 0.01
*Bladder cancer-specific items*																	
I have control of my bowels	14	13.3	91	86.7	30	15.3	166	84.7	27	20.3	106	79.7	45	25.0	135	75.0	*χ*^2^ = 8.3, *p* = 0.04
I urinate more frequently than usual	73	70.9	30	29.1	117	63.6	67	36.4	87	70.7	36	29.3	99	55.6	79	44.4	*χ*^2^ = 10, *p* = 0.019
I have a good appetite	13	12.1	94	87.9	31	16.0	163	84.0	21	15.8	112	84.2	50	26.9	136	73.1	*χ*^2^ = 13, *p* < 0.01
It burns when I urinate	99	98.0	2	2.0	174	92.6	14	7.4	120	94.5	7	5.5	153	89.0	19	11.0	*χ*^2^ = 8.5, *p* = 0.036
I am interested in sex	52	54.2	44	45.8	98	58.0	71	42.0	82	69.5	36	30.5	130	77.8	37	22.2	*χ*^2^ = 21.8, *p* < 0.01
(For men only) I am able to have and maintain an erection	42	61.8	26	38.2	87	66.4	44	33.6	75	78.1	21	21.9	107	82.9	22	17.1	*χ*^2^ = 15.1, *p* < 0.01

#### Social/family well-being

Of the 51% who answered this item, two thirds (67%) reported dissatisfaction with their sex life ('not at all' or 'a little' satisfied with their sex life). Dissatisfaction was significantly more likely to be reported by patients who underwent RC surgery compared to those who had other treatments (Table 4). A higher percentage of females reported that they were 'quite a bit' or 'very much' satisfied with their sex life (51% compared to 31% of males, *p* < 0.01).

#### Emotional well-being

Respondents across the cohort reported a lack of satisfaction with how they were coping with their illness, as almost three-quarters of respondents reported that they were 'not at all' or 'a little' satisfied. Feeling 'quite a bit' or 'very much' nervous was reported by 10% of respondents; particularly by females (18% compared to 7% of males, *p* < 0.01), and those with ≥3 LTCs (Table 5).

#### Functional well-being

Around a third of respondents (35%) answered 'not at all' or 'a little' about their ability to work. Respondents treated with radiotherapy were less likely to be able to work compared to respondents receiving other treatments (Table 4).

Although three quarters of respondents reported that they were content with the quality of their life right now (reporting 'quite a bit' or 'very much'), respondents with ≥3 LTCs were significantly more likely to report that they were not content (Table 5).

#### Bladder cancer-specific items

##### Urinary items

Urinating more frequently than usual was common after endoscopy (reported 'quite a bit' or 'very much' in 34–37%) and radiotherapy (reported 'quite a bit' or 'very much' in 44–50%) (Table 4).

##### Sexual items

Disinterest in sex was reported by 66% of respondents and had a good response rate of 85%. Disinterest in sex was significantly higher in females than males, with 86% of females saying they were 'not at all' or only 'a little' interested in sex, compared to 60% of males (*p* < 0.01). This difference was observed (but not significant due to small numbers) when restricted to those who had a stoma, with 89% of females saying they were 'not at all' or only 'a little' interested. Ability to maintain an erection was less likely in males who had a stoma, with 96% reporting 'not at all' or 'a little' to this item, though the result was not significant due to small numbers.

##### Body image

Just under half of respondents said that they didn’t like their body appearance at all, or only liked it a little (48%). Respondents with a stoma were more likely to report not liking their body at all or only liking it a little (60% compared to 46% of respondents without a stoma, *p* = 0.01).

## Discussion

Here we report HRQL in individuals between 1 and 5 years post diagnosis for BC. While modest in size compared to PROMs studies in other cancer sites, this work represents the largest UK study to date and demonstrates this methodology is feasible in this population. We have identified that reduced HRQL is common in patients following BC treatment, that there are differences according to treatment modality and patient characteristics, and that further more focused studies are warranted.

Several key findings deserve discussion. First, our results highlight the need to support people who have pre-existing health conditions and a new diagnosis of BC. Respondents with LTCs were much more likely to report poor HRQL across all EQ-5D-5L items, all domains apart from Money Matters on the SDI-21, SD-16 and on multiple items of FACT-Bl. The design and methodology used in this survey limits our ability to investigate this further and to understand whether this reflects the impact of BC on other LTCs, or the impact of other LTCs on HRQL. This is an important area for future studies to focus on.

Second, while we do not know details of each tumour (i.e., stage or grade), most patients (60%) received only endoscopic surgery. To date, most BC HRQL reports have focused upon MIBC and cystectomy outcomes. As such, our data are the first to look at HRQL in MIBC and NMIBC outcomes across a UK population. When comparing NMIBC treatments, overall, respondents receiving endoscopic surgery with intravesical chemotherapy had higher HRQL and fewer everyday living difficulties than those receiving only endoscopic surgery. This may reflect recall bias (guidelines suggest that most patients should have received intravesical chemotherapy),^4^ performance status (unfit patients did not receive intravesical chemotherapy), treatment differences (intravesical chemotherapy improves disease outcomes) or service design (perhaps better designed services are more guideline compliant and more likely to support patients through treatment). Support for patient selection has shown that for many domains the HRQL was superior for combined treatment rather than just endoscopic surgery.

Third, around 30% of respondents received radical therapy, including 16% who had a stoma and 9% who had received radiotherapy. The latter were most likely to report low HRQL, problems with mobility, self-care and usual activities. They were also more likely to be socially distressed (score ≥10 on SD-16), have high levels of difficulty with everyday living, report a lack of energy and an inability to work. Patients treated with radiotherapy were also more likely to report needing to urinate more frequently than usual. While these findings may reflect outcomes from radiotherapy, when compared to RC, it is more likely they reveal treatment patterns and pre-existing fitness.^26^ Evidence to support this is that most of these measures were better for patients who received both radiotherapy with chemotherapy (for which higher fitness is needed). Indeed outcomes from RC and radiotherapy with chemotherapy were broadly comparable to each other and to patients receiving only endoscopy/telescopy.^27^ Finally, overall there were some encouraging findings with social distress generally being low in respondents, as 85% were below the cutoff point, and perfect health (i.e., no problems on EQ-5D-5L) was reported by 35% of respondents.

This study has a number of key limitations. Response rates were marginally lower than for UK surveys in other cancer sites (63% for colorectal cancer)^14^ and 68% overall for a pilot study of individuals diagnosed with non-Hodgkin’s lymphoma (62%), breast (68%), colorectal (64%) and prostate cancer (69%)).^17^ This may reflect the BC population (i.e., typically more deprived, more manual workers and lower literacy rates than other cancers).^28^ While respondents were willing to answer personal questions, response rates for sexual items were lower than for other domains. Details of disease stage were not available and treatment details were self-reported (and not verified from other sources) thereby reducing ability to interpret data in detail.

A major limitation was the removal by the survey developers of the 'somewhat' response option from the FACT-Bl questionnaire, which meant that composite scores could not be calculated, thus affecting the interpretation of results. Although the removal of response options from validated measures is not considered good measurement practice, we were still able to gain important information about patients who had few or no problems and patients who had severe problems with individual items. Despite this limitation, it was considered important to present the findings as there is a lack of large-scale studies looking at all BC populations. A further limitation was that, as it was a pilot study, the sample was randomly identified, rather than population-based.

The results have been presented descriptively and multivariable analysis was not undertaken. The small number of respondents in some subgroups (e.g., in some of the treatment groups) and the lack of information on important confounders (such as a measure of socioeconomic deprivation) make it difficult to obtain robust, meaningful results.

Although more detailed analysis could not be carried out in this study, it is important that future studies aim to incorporate this. In particular, quantifying the impact of treatment-related issues (e.g., urinary, bowel, sexual problems or fatigue) on HRQL and social distress is hugely important, as this will further highlight the support and care needs of this group of patients, and indicate where there are gaps in service provision.

Recent qualitative work highlighted gaps in the understanding of HRQL of BC patients (particularly patients with NMIBC).^12^ Important themes included post-treatment experiences in terms of family/friend support networks, dealing with incontinence, voiding and catheterising, the 'new normal' (e.g., coping with their post-surgery body), changing sexuality and living with the lifelong threat of cancer.^12^ Although the authors recommend longitudinal qualitative work with BC patients, based on the results of the DH study, there is also a need to undertake quantitative work to understand how HRQL changes in BC patients over time. Future work should aim to identify both high risk groups and treatment-related items with the biggest impact on HRQL. This could potentially lead to PROMs being used as part of routine practice, with risk factors for low HRQL monitored in clinic.

A further recommendation for future BC PROMs work using FACT-Bl is to include some validation work within the analysis. Although FACT-G is widely considered to be a reliable and valid tool to use with cancer patients, the bladder cancer-specific items require psychometric analysis to understand how useful these items are for use with BC populations. Alternatively, clinicians and researchers may choose other BC specific measures, such as the Bladder Cancer Index (BCI),^29^ or the European Organisation for Research and Treatment of Cancer (EORTC) NMIBC and MIBC modules.^30,31^

These data represent the largest PROMs study to use BC specific PROMs. The results have highlighted groups at high risk of significant adverse consequences following BC diagnosis. However, there is a need to carry out larger in-depth population-based HRQL studies of BC patients to fully understand the extent of the morbidity burden experienced by survivors of BC.

## Electronic supplementary material

Supplementary Table 1. Number of Respondents Receiving Each Treatment Type by Demographic

Supplementary Table 2. Comparison of Demographics and Clinical Characteristics Between Respondents and Nonrespondents

Supplementary Table 3. Missing Responses Data by Question

Supplementary File 1

## Availability of data and material

The aggregated results of data analysis, STATA files and output for data extraction are available from the authors on request. Individual patient-level data used to generate results are not freely available, but may be applied for through the PHE Office for Data Release (ODR).
