# Supplementary material for: Health-related quality of life after treatment for bladder cancer in England
Source: Br J Cancer. 2018 May 14;118(11):1518–28. doi: 10.1038/s41416-018-0084-z (PMC5988662; doi:10.1038/s41416-018-0084-z)
Supplement: Supplementary file 1 — Supplementary Table 1. Number of Respondents Receiving Each Treatment Type by Demographic [file 41416_2018_84_MOESM1_ESM.docx]

| **Supplementary Table 1. Number of Respondents Receiving Each Treatment Type by Demographic** | | | | | | | |  |  |  |  |
| --- | --- | --- | --- | --- | --- | --- | --- | --- | --- | --- | --- |
| **Demographic** | **Endoscopy/telescopy** | | **Endoscopy/telescopy with chemotherapy directly into the bladder** | | **Radical Cystectomy** | | **Radiotherapy and Intravenous chemotherapy** | | **Radiotherapy** | | **p** |
|  | **N** | **%** | **N** | **%** | **N** | **%** | **N** | **%** | **N** | **%** |  |
| **Age, years** | | | | | | | | | | | |
| <55 | 11 | 23.9 | 18 | 39.1 | 12 | 26.1 | 5 | 10.9 | 0 | 0.0 | NA |
| 55-64 | 32 | 27.8 | 49 | 42.6 | 27 | 23.5 | 4 | 3.5 | 3 | 2.6 |  |
| 65-74 | 76 | 34.2 | 74 | 33.3 | 33 | 14.9 | 23 | 10.4 | 16 | 7.2 |  |
| 75-84 | 65 | 38.7 | 51 | 30.4 | 17 | 10.1 | 4 | 2.4 | 31 | 18.5 |  |
| ≥ 85 | 17 | 53.1 | 4 | 12.5 | 1 | 3.1 | 0 | 0.0 | 10 | 31.3 |  |
| **Sex** | | | | | | | | | | | |
| Male | 169 | 38.3 | 142 | 32.2 | 61 | 13.8 | 24 | 5.4 | 45 | 10.2 | χ2=13.4, p<0.01 |
| Female | 33 | 22.6 | 54 | 37.0 | 30 | 20.6 | 12 | 8.2 | 17 | 11.6 |  |
| **No. of long-term conditions** | | | | | | | | | | | |
| None | 29 | 28.7 | 40 | 39.6 | 18 | 17.8 | 8 | 7.9 | 6 | 5.9 | χ2=14.2, p=0.285 |
| 1 | 56 | 30.9 | 61 | 33.7 | 32 | 17.7 | 12 | 6.6 | 20 | 11.1 |  |
| 2 | 41 | 33.9 | 40 | 33.1 | 21 | 17.4 | 5 | 4.1 | 14 | 11.6 |  |
| ≥ 3 | 72 | 41.1 | 52 | 29.7 | 18 | 10.3 | 11 | 6.3 | 22 | 12.6 |  |
