# Supplementary material for: Health-related quality of life after treatment for bladder cancer in England
Source: Br J Cancer. 2018 May 14;118(11):1518–28. doi: 10.1038/s41416-018-0084-z (PMC5988662; doi:10.1038/s41416-018-0084-z)
Supplement: Supplementary file 2 — Supplementary Table 2. Comparison of Demographics and Clinical Characteristics Between Respondents and Nonrespondents [file 41416_2018_84_MOESM2_ESM.docx]

| **Supplementary Table 2. Comparison of Demographics and Clinical Characteristics Between Respondents and Nonrespondents** | | | | | | |
| --- | --- | --- | --- | --- | --- | --- |
| **Demographic or clinical characteristic** | **Non-respondents** | | **Respondents** | | **Total** | **Response Rate (%)** |
|  | **N** | **%** | **N** | **%** |  |  |
| **Sex** | | | | | | |
| Male | 413 | 71.5% | 508 | 75.5% | 921 | 55.2 |
| Female | 165 | 28.5% | 165 | 24.5% | 330 | 50.0 |
| Total | 578 | 100.0% | 673 | 100.0% | 1251 | 53.8 |
| **Age** |  |  |  |  |  |  |
| <55 | 44 | 7.6% | 47 | 7.0% | 91 | 51.6 |
| 55-64 | 91 | 15.7% | 128 | 19.0% | 219 | 58.4 |
| 65-74 | 164 | 28.4% | 252 | 37.4% | 416 | 60.6 |
| 75-84 | 198 | 34.3% | 194 | 28.8% | 392 | 49.5 |
| 85+ | 81 | 14.0% | 52 | 7.7% | 133 | 39.1 |
| Total | 578 | 100.0% | 673 | 100.0% | 1251 | 53.8 |
| **Stage** |  |  |  |  |  |  |
| 1 | 42 | 7.3% | 73 | 10.8% | 115 | 63.5 |
| 2 | 15 | 2.6% | 23 | 3.4% | 38 | 60.5 |
| 3 | 6 | 1.0% | 3 | 0.4% | 9 | 33.3 |
| 4 | 3 | 0.5% | 6 | 0.9% | 9 | 66.7 |
| Unknown | 512 | 88.6% | 568 | 84.4% | 1080 | 52.6 |
| Total | 578 | 100% | 673 | 100.0% | 1251 | 53.8 |
| NOTE. Based on cancer registry data available for all individuals | | | | | | |
