# Supplementary material for: Health-related quality of life after treatment for bladder cancer in England
Source: Br J Cancer. 2018 May 14;118(11):1518–28. doi: 10.1038/s41416-018-0084-z (PMC5988662; doi:10.1038/s41416-018-0084-z)
Supplement: Supplementary file 3 — Supplementary Table 3. Missing Responses Data by Question [file 41416_2018_84_MOESM3_ESM.docx]

| **Supplementary Table 3. Missing Responses Data by Question** | | | | | |
| --- | --- | --- | --- | --- | --- |
| **Question No.** | **Description** | **No. of Responses** | **Total No. of Respondents** | **Missing Responses** | |
|  |  |  |  | **No.** | **%** |
| 1 | Treatment | 644 | 673 | 29 | 4.3 |
| 3 | Disease status | 593 | 673 | 80 | 11.9 |
| 4 - 8 | EQ-5D all domains | 639 | 673 | 34 | 5.1 |
| 9 -15 | FACT-Bl Physical wellbeing | 594 - 635 | 673 | 38 - 79 | 5.6 - 11.7 |
| 16 -21 | FACT-Bl Social/family wellbeing | 572 - 598 | 673 | 75 - 101 | 11.1 - 15.0 |
| 22 | FACT-Bl Social/family wellbeing - satisfied with sex life | 345 | 673 | 328 | 48.7 |
| 23 -27 | FACT-Bl Emotional wellbeing | 420 - 454 | 673 | 219 - 253 | 32.5 - 37.6 |
| 28 | FACT-Bl Emotional wellbeing - I worry that my condition will get worse | 625 | 673 | 48 | 7.1 |
| 29-35 | FACT-Bl Functional wellbeing | 549 - 633 | 673 | 40 - 124 | 5.9 - 18.4 |
| 36 | FACT-Bl Bladder cancer specific items - I have trouble controlling my urine | 622 | 673 | 51 | 7.6 |
| 37 | FACT-Bl Bladder cancer specific items - I am losing weight | 609 | 673 | 64 | 9.5 |
| 38 | FACT-Bl Bladder cancer specific items - I have control of my bowels | 635 | 673 | 38 | 5.6 |
| 39 | FACT-Bl Bladder cancer specific items - I urinate more frequently than usual | 607 | 673 | 66 | 9.8 |
| 40 | FACT-Bl Bladder cancer specific items - I have diarrhoea | 621 | 673 | 52 | 7.7 |
| 41 | FACT-Bl Bladder cancer specific items - I have a good appetite | 642 | 673 | 31 | 4.6 |
| 42 | FACT-Bl Bladder cancer specific items - I like the appearance of my body | 586 | 673 | 87 | 12.9 |
| 43 | FACT-Bl Bladder cancer specific items - it burns when I urinate | 607 | 673 | 66 | 9.8 |
| 44 | FACT-Bl Bladder cancer specific items - I am interested in sex | 570 | 673 | 103 | 15.3 |
| 45 | FACT-Bl Bladder cancer specific items - I am able to have or maintain an erection (men only) | 430 | 500 | 70 | 14.0 |
| 46 | FACT-Bl Bladder cancer specific items - Stoma | 545 | 673 | 128 | 19.0 |
| 50 - 53, 66, 68 | SDI Everyday Living Scale | 620 - 653 | 673 | 20 - 53 | 3.0 - 7.9 |
| 55 - 59 | SDI Money Matters Scale | 600 - 641 | 673 | 32 - 73 | 4.8 - 10.8 |
| 60 - 61, 64 - 65, 67, 69 - 70 | SDI Self and Others Scale | 620 - 651 | 673 | 22 - 53 | 3.3 - 7.9 |
| 62 | SDI - Sexual matters | 594 | 673 | 79 | 11.7 |
| 63 | SDI - Plans to have a family | 605 | 673 | 68 | 10.1 |
| 67 | SDI - Where you live | 652 | 673 | 21 | 3.1 |
| 69 | SDI - Holidays | 647 | 673 | 26 | 3.9 |
| 70 | SDI - Other | 637 | 673 | 36 | 5.3 |
| 91 | Gender | 662 | 673 | 11 | 1.6 |
| 96 | LTCs | 650 | 673 | 23 | 3.4 |
| 100 | Ethnicity | 673 | 673 | 0 | 0.0 |
