# Supplementary material for: Health-related quality of life after treatment for bladder cancer in England
Source: Br J Cancer. 2018 May 14;118(11):1518–28. doi: 10.1038/s41416-018-0084-z (PMC5988662; doi:10.1038/s41416-018-0084-z)
Supplement: Supplementary file 4 — Supplementary File 1 [file 41416_2018_84_MOESM4_ESM.pdf]

picker Institute Europe

Making patients' views count

NHS

Living with and Beyond Bladder Cancer

More people are now living after a diagnosis and treatment for cancer. But we do not know enough about what life is *really* like for you.

We are collecting information about the health and wellbeing of patients who have had a diagnosis of bladder cancer, in order to help inform our understanding and improve the quality of services for cancer patients in the future. We would therefore be grateful for information on your health and quality of life.

On rare occasions, although our records suggest that you had a diagnosis of cancer, it was only because it had been suspected but was then not confirmed. So, if this questionnaire is not relevant to you, please accept our apologies, get in touch with our help line or just return the blank questionnaire, and we will correct our records.

The information you give us will be treated **in confidence** and we will not publish any personal information that could allow anyone to identify you.

Instructions to help you complete this form can be found on the inside front cover.

We are very grateful for your time and effort in completing this survey. If you have any queries about the questionnaire, please call the FREEPHONE helpline number: 0800 783 2896

You can find more information about this and other surveys at: cancerproms.ncr.nhs.uk

Please return this questionnaire, in the envelope provided, to:
Freepost Plus RSHK-XBRS-RKRJ, Picker Institute Europe,
10 Warboys Airfield Industrial Estate, Warboys, HUNTINGDON, PE28 2SH

IMPORTANT INFORMATION

The purpose of this questionnaire is to collect information about the quality of healthcare services. The information collected will be used to produce statistics about the quality of healthcare services offered by different providers (hospitals) across the NHS. These statistics will be used to measure and improve the quality of healthcare services.

With your permission, the personal details that you provide, and other information held about you in other NHS databases, will be used to analyse and interpret the information collected.

By completing this questionnaire you are giving your consent for the information provided to be used for the purposes set out above. You are agreeing that:

- Your personal details, and other relevant information related to you, will be held and used by the National Cancer Registration Service and the Health and Social Care Information Centre, including relevant information held about you by the Personal Demographics Service, the Demographics Batch Service, the Secondary Uses Service and other NHS databases.
- Your personal details can be used to send to you related follow up questionnaires in the future.
- Your personal details and health information can be held by contractors working on behalf of the National Cancer Registration Service, Health and Social Care Information Centre and Department of Health.

Your personal information will be handled securely and will be stored as part of the patient cancer record by the National Cancer Registration Service. The information from this questionnaire may be shared with healthcare professionals involved in your case under the strict data handling rules for patient identifiable information.

Information from this questionnaire may also be stored by the Health and Social Care Information Centre. The National Cancer Registration Service, the Health and Social Care Information Centre, the Department of Health and contractors working on their behalf will not release your personal information, unless required by law, or where there is a clear overriding public interest.

Your participation is voluntary. If you do not want to take part, do not fill in the questionnaire. You may withdraw the information you give the NHS in this questionnaire upon request, up to the point at which the data are analysed and personal details removed.

Who should complete the questionnaire?

The questions should be answered by the person named in the letter that came with this questionnaire. If that person needs help to answer the questions then the answers should be given from their point of view – not from the point of view of the person who is helping.

Completing the questionnaire

Please use a black or blue pen and for each question tick clearly inside the box that best represents your views.

Do not worry if you make a mistake. Just cross out the mistake and put a tick in the correct box.

You do not need to write your name or address anywhere on the questionnaire.

The more questions in this survey that you complete, the more we can understand what life is like for those living with and beyond cancer. However, if you feel unable or uncomfortable about answering any of the questions, leave it blank and move on to the next one.

This page has been left intentionally blank

YOUR HEALTH TODAY

The questions in this section are all about your health and how you feel on the day you complete this questionnaire.

1. What treatments have you received for your bladder cancer? **Tick all that apply.**

1 ☐ Telescopic/endoscopic bladder tumour surgery
2 ☐ Radical cystectomy (removal of the bladder)
3 ☐ Chemotherapy into the bladder
4 ☐ Intravenous chemotherapy (ie, directly into a vein)
5 ☐ Radiotherapy

2. How long is it since you completed your **initial treatment** for bladder cancer? (Treatment includes any chemotherapy, radiotherapy, or surgery for your bladder cancer. When answering this question please do not include hormone treatments.)

1 ☐ I am still having my initial treatment
2 ☐ It is less than 3 months since my initial treatment
3 ☐ It is between 3 and 12 months since my initial treatment
4 ☐ It is between 1 and 5 years since my initial treatment
5 ☐ It is more than 5 years since my initial treatment
6 ☐ Don't know/ can't remember

3. How has your bladder cancer responded to treatment?

1 ☐ My bladder cancer has responded fully to treatment (I am in remission)
2 ☐ My bladder cancer has been treated but is still present
3 ☐ My bladder cancer has not been treated at all
4 ☐ My bladder cancer has come back after it was originally treated
5 ☐ I am not certain what is happening with my bladder cancer

YOUR HEALTH TODAY

Under each heading, please tick the ONE box that best describes your health TODAY.

4. MOBILITY

I have no problems in walking about	☐ 1
I have slight problems in walking about	☐ 2
I have moderate problems in walking about	☐ 3
I have severe problems in walking about	☐ 4
I am unable to walk about	☐ 5

5. SELF CARE

I have no problems washing or dressing myself	☐ 1
I have slight problems washing or dressing myself	☐ 2
I have moderate problems washing or dressing myself	☐ 3
I have severe problems washing or dressing myself	☐ 4
I am unable to wash or dress myself	☐ 5

6. USUAL ACTIVITIES (work, study, housework, family or leisure activities)

I have no problems doing my usual activities	☐ 1
I have slight problems doing my usual activities	☐ 2
I have moderate problems doing my usual activities	☐ 3
I have severe problems doing my usual activities	☐ 4
I am unable to do my usual activities	☐ 5

7. PAIN/ DISCOMFORT

I have no pain or discomfort	☐ 1
I have slight pain or discomfort	☐ 2
I have moderate pain or discomfort	☐ 3
I have severe pain or discomfort	☐ 4
I have extreme pain or discomfort	☐ 5

8. ANXIETY/ DEPRESSION

I am not anxious or depressed	☐ 1
I am slightly anxious or depressed	☐ 2
I am moderately anxious or depressed	☐ 3
I am severely anxious or depressed	☐ 4
I am extremely anxious or depressed	☐ 5

YOUR HEALTH AND WELLBEING IN THE PAST WEEK

The questions in this section are about your health and wellbeing and how you have felt in the past WEEK.

Please mark one box per line to give your response.

	**During the past week**	**Not at all**	**A little bit**	**Quite a bit**	**Very much**
**9.**	I have a lack of energy	1 ☐	2 ☐	3 ☐	4 ☐
**10.**	I have nausea	1 ☐	2 ☐	3 ☐	4 ☐
**11.**	Because of my physical condition, I have trouble meeting the needs of my family	1 ☐	2 ☐	3 ☐	4 ☐
**12.**	I have pain	1 ☐	2 ☐	3 ☐	4 ☐
**13.**	I am bothered by side effects of the treatment	1 ☐	2 ☐	3 ☐	4 ☐
**14.**	I feel ill	1 ☐	2 ☐	3 ☐	4 ☐
**15.**	I am forced to spend time in bed	1 ☐	2 ☐	3 ☐	4 ☐
**16.**	I feel close to my friends	1 ☐	2 ☐	3 ☐	4 ☐
**17.**	I get emotional support from my family	1 ☐	2 ☐	3 ☐	4 ☐
**18.**	I get support from my friends	1 ☐	2 ☐	3 ☐	4 ☐
**19.**	My family has accepted my illness	1 ☐	2 ☐	3 ☐	4 ☐
**20.**	I am satisfied with family communications about my illness	1 ☐	2 ☐	3 ☐	4 ☐
**21.**	I feel close to my partner (or the person who is my main support)	1 ☐	2 ☐	3 ☐	4 ☐
	Regardless of your current level of sexual activity, please answer the following question. If you prefer not to answer it, please mark this box.	1 ☐			
**22.**	I am satisfied with my sex life	1 ☐	2 ☐	3 ☐	4 ☐
**23.**	I feel sad	1 ☐	2 ☐	3 ☐	4 ☐
**24.**	I am satisfied with how I am coping with my illness	1 ☐	2 ☐	3 ☐	4 ☐
**25.**	I am losing hope in the fight against my illness	1 ☐	2 ☐	3 ☐	4 ☐
**26.**	I feel nervous	1 ☐	2 ☐	3 ☐	4 ☐
**27.**	I worry about dying	1 ☐	2 ☐	3 ☐	4 ☐

The questions in this section are about your health and wellbeing and how you have felt in the past WEEK.

Please mark one box per line to give your response.

	**During the past week**	**Not at all**	**A little bit**	**Quite a bit**	**Very much**
**28.**	I worry that my condition will get worse	1 ☐	2 ☐	3 ☐	4 ☐
**29.**	I am able to work (include work at home)	1 ☐	2 ☐	3 ☐	4 ☐
**30.**	My work (include work at home) is fulfilling	1 ☐	2 ☐	3 ☐	4 ☐
**31.**	I am able to enjoy life	1 ☐	2 ☐	3 ☐	4 ☐
**32.**	I have accepted my illness	1 ☐	2 ☐	3 ☐	4 ☐
**33.**	I am sleeping well	1 ☐	2 ☐	3 ☐	4 ☐
**34.**	I am enjoying the things I usually do for fun	1 ☐	2 ☐	3 ☐	4 ☐
**35.**	I am content with the quality of my life right now	1 ☐	2 ☐	3 ☐	4 ☐
**36.**	I have trouble controlling my urine	1 ☐	2 ☐	3 ☐	4 ☐
**37.**	I am losing weight	1 ☐	2 ☐	3 ☐	4 ☐
**38.**	I have control of my bowels	1 ☐	2 ☐	3 ☐	4 ☐
**39.**	I urinate more frequently than usual	1 ☐	2 ☐	3 ☐	4 ☐
**40.**	I have diarrhoea	1 ☐	2 ☐	3 ☐	4 ☐
**41.**	I have a good appetite	1 ☐	2 ☐	3 ☐	4 ☐
**42.**	I like the appearance of my body	1 ☐	2 ☐	3 ☐	4 ☐
**43.**	It burns when I urinate	1 ☐	2 ☐	3 ☐	4 ☐
**44.**	I am interested in sex	1 ☐	2 ☐	3 ☐	4 ☐
**45.**	(For men only) I am able to have and maintain an erection	1 ☐	2 ☐	3 ☐	4 ☐
**46.**	Do you have a stoma appliance? If yes, please answer the following two items:	No 1 ☐		Yes 2 ☐	
**47.**	I am embarrassed by my stoma appliance	1 ☐	2 ☐	3 ☐	4 ☐
**48.**	Caring for my stoma appliance is difficult	1 ☐	2 ☐	3 ☐	4 ☐

49. In the **past week**, on how many days have you done a total of 30 minutes or more of physical activity, which was enough to raise your heart rate? *This may include sport, exercise and brisk walking or cycling for recreation or to get to and from places, but should not include housework or physical activity that is part of your job.*

**None**	**1 day**	**2 days**	**3 days**	**4 days**	**5 days**	**6 days**	**7 days**
0 ☐	1 ☐	2 ☐	3 ☐	4 ☐	5 ☐	6 ☐	7 ☐

YOUR HEALTH AND WELLBEING IN THE PAST MONTH

The questions in this section are about your health and wellbeing and how you have felt in the past MONTH.

Sometimes people who have, or have had, cancer find that they have a number of everyday difficulties to cope with following their diagnosis. These might be to do with things like their family life, social activities, finances and work.

Please tick the response that best describes your answer.

	During the past month	No difficulty	A little	Quite a bit	Very much	Does not apply
**50.**	Have you had any difficulty in maintaining your independence?	1 ☐	2 ☐	3 ☐	4 ☐	5 ☐
**51.**	Have you had any difficulty in carrying out your domestic chores? (e.g. cleaning, gardening, cooking, shopping)	1 ☐	2 ☐	3 ☐	4 ☐	5 ☐
**52.**	Have you had any difficulty with managing your own personal care? (e.g. bathing, dressing, washing)	1 ☐	2 ☐	3 ☐	4 ☐	5 ☐
**53.**	Have you had any difficulty with looking after those who depend on you? (e.g. children, dependent adults, pets)	1 ☐	2 ☐	3 ☐	4 ☐	5 ☐
**54.**	Have any of those close to you (e.g. partner, children, parents) had any difficulty with the support available to them?	1 ☐	2 ☐	3 ☐	4 ☐	5 ☐
**55.**	Have you had any difficulty with benefits? (e.g. statutory sick pay, attendance allowance, disability living allowance)	1 ☐	2 ☐	3 ☐	4 ☐	5 ☐
**56.**	Have you had any financial difficulties?	1 ☐	2 ☐	3 ☐	4 ☐	5 ☐
**57.**	Have you had any difficulty with financial services? (e.g. loans, mortgages, pensions, insurance)	1 ☐	2 ☐	3 ☐	4 ☐	5 ☐
**58.**	Have you had any difficulty concerning your work? (or education if you are a student)	1 ☐	2 ☐	3 ☐	4 ☐	5 ☐
**59.**	Have you had any difficulty with planning for your own or your family's future? (e.g. care of dependents, legal issues, business affairs)	1 ☐	2 ☐	3 ☐	4 ☐	5 ☐

YOUR HEALTH AND WELLBEING IN THE PAST MONTH

The questions in this section are about your health and wellbeing and how you have felt in the past MONTH.

Please tick the response that best describes your answer.

	**During the past month**	**No difficulty**	**A little**	**Quite a bit**	**Very much**	**Does not apply**
**60.**	Have you had any difficulty with communicating with those closest to you? (e.g. partner, children, parents)	1 ☐	2 ☐	3 ☐	4 ☐	5 ☐
**61.**	Have you had any difficulty with communicating with others? (e.g. friends, neighbours, colleagues, dates)	1 ☐	2 ☐	3 ☐	4 ☐	5 ☐
**62.**	Have you had any difficulty concerning sexual matters?	1 ☐	2 ☐	3 ☐	4 ☐	5 ☐
**63.**	Have you had any difficulty concerning plans to have a family?	1 ☐	2 ☐	3 ☐	4 ☐	5 ☐
**64.**	Have you had any difficulty concerning your appearance or body image?	1 ☐	2 ☐	3 ☐	4 ☐	5 ☐
**65.**	Have you felt isolated?	1 ☐	2 ☐	3 ☐	4 ☐	5 ☐
**66.**	Have you had any difficulty with getting around? (e.g. transport, car parking, your mobility)	1 ☐	2 ☐	3 ☐	4 ☐	5 ☐
**67.**	Have you had any difficulty with where you live? (e.g. space, access, damp, heating, neighbours, security)	1 ☐	2 ☐	3 ☐	4 ☐	5 ☐
**68.**	Have you had any difficulty in carrying out your recreational activities? (e.g. hobbies, pastimes, social pursuits)	1 ☐	2 ☐	3 ☐	4 ☐	5 ☐
**69.**	Have you had any difficulty with your plans to travel or take a holiday?	1 ☐	2 ☐	3 ☐	4 ☐	5 ☐
**70.**	Have you had any difficulty with any other area of your everyday life?	1 ☐	2 ☐	3 ☐	4 ☐	5 ☐

YOUR HEALTH AND WELLBEING IN THE PAST MONTH

The questions in this section are about your health and wellbeing and how you have felt in the past MONTH.

Please tick the response that best describes your answer.

	During the past month	Strongly agree	Agree	Neither agree nor disagree	Disagree	Strongly disagree	Does not apply to me
**71.**	I have fears about my cancer spreading	1 ☐	2 ☐	3 ☐	4 ☐	5 ☐	6 ☐
**72.**	I have fears about my cancer coming back	1 ☐	2 ☐	3 ☐	4 ☐	5 ☐	6 ☐
**73.**	I have fears about death and dying	1 ☐	2 ☐	3 ☐	4 ☐	5 ☐	6 ☐
**74.**	I experience memory loss	1 ☐	2 ☐	3 ☐	4 ☐	5 ☐	6 ☐
**75.**	I have trouble sleeping	1 ☐	2 ☐	3 ☐	4 ☐	5 ☐	6 ☐
**76.**	I have trouble concentrating	1 ☐	2 ☐	3 ☐	4 ☐	5 ☐	6 ☐
**77.**	I always feel tired	1 ☐	2 ☐	3 ☐	4 ☐	5 ☐	6 ☐
**78.**	I experience mood swings	1 ☐	2 ☐	3 ☐	4 ☐	5 ☐	6 ☐
**79.**	I am often irritable	1 ☐	2 ☐	3 ☐	4 ☐	5 ☐	6 ☐

OVERALL SUPPORT AND CARE

80. Do you have an up to date written care plan? (A care plan is a document that sets out your needs and goals for caring for your cancer.)

1 ☐ Yes, definitely
2 ☐ Yes, I think so
3 ☐ No
4 ☐ I do not need a care plan
5 ☐ Don't know

81. Do you have a named nurse who you can contact if you have a worry about your cancer care? (A named nurse is sometimes known as a Clinical Nurse Specialist or Specialist Cancer Nurse.)

1 ☐ Yes
2 ☐ No
3 ☐ Don't know

82. Do you know who to contact if you have a concern about any aspect of living with or after cancer?

1 ☐ Yes, definitely
2 ☐ Yes, I think so
3 ☐ No

83. Do you think that hospital staff did everything they could to support you following your cancer treatment?

1 ☐ Yes, all of the time
2 ☐ Only some of the time
3 ☐ Never
4 ☐ I did not need any support

84. Do you think that GPs and nurses at your general practice do everything they can to support you following your cancer treatment?

1 ☐ Yes, all of the time
2 ☐ Only some of the time
3 ☐ Never
4 ☐ My general practice is not involved
5 ☐ I do not need any support

85. Following your initial cancer treatment have you been given enough care and help from health and social services (for example, district nurses, home helps or occupational therapists)?

1 ☐ Yes, definitely
2 ☐ Yes, to some extent
3 ☐ No
4 ☐ I did not need help from health or social services
5 ☐ Don't know/ can't remember

86. Do you consider yourself to be a

1 ☐ Smoker
2 ☐ Ex-smoker
3 ☐ Non-smoker

87. If an ex-smoker, how long ago did you stop?

1 ☐ less than 1 year
2 ☐ less than 2 years
3 ☐ less than 3 years
4 ☐ less than 4 years
5 ☐ less than 5 years
6 ☐ more than 5 years

88. Did you receive any advice or information on any of the following issues? **Tick all that apply.**

1 ☐ Diet and lifestyle (including stopping smoking)

2 ☐ Physical activity and exercise

3 ☐ Financial help or benefits

4 ☐ Free prescriptions

5 ☐ Returning to or staying in work

6 ☐ Information/ advice for family/ friends/ carer

7 ☐ The physical aspects of living with and after cancer (e.g. side effects or signs of recurrence)

8 ☐ The psychological or emotional aspects of living with and after cancer

9 ☐ How to access support groups

10 ☐ I have all the information and advice I need

11 ☐ I was not offered any of the above

89. Would it have been helpful to have had more advice or information on any of the following issues? **Tick all that apply.**

1 ☐ Diet and lifestyle (including stopping smoking)

2 ☐ Physical activity and exercise

3 ☐ Financial help or benefits

4 ☐ Free prescriptions

5 ☐ Returning to or staying in work

6 ☐ Information/ advice for family/ friends/ carer

7 ☐ The physical aspects of living with and after cancer (e.g. side effects or signs of recurrence)

8 ☐ The psychological or emotional aspects of living with and after cancer

9 ☐ How to access support groups

10 ☐ I have all the information and advice I need

ABOUT YOU

If you are helping someone to complete this questionnaire, please make sure this information is the patient's and not your own.

90. What **year** were you born?

Please write in e.g. | 1 | 9 | 4 | 4 |

Y	Y	Y	Y

91. Are you male or female?

1 ☐ Male
2 ☐ Female

92. Do you look after, or give any help or support (not as part of your paid employment) to family members, friends, neighbours or others because of either:

- Long term physical or mental health/ disability, or
- Problems relating to old age

1 ☐ Yes
2 ☐ No

93. Which of the following best describes your sexual orientation?

1 ☐ Heterosexual/ straight (opposite sex)
2 ☐ Bisexual (both sexes)
3 ☐ Gay (same sex)
4 ☐ Other
5 ☐ Prefer not to answer

94. Which statement best describes your living arrangements?

1 ☐ I live with partner/ spouse/ family/ friends
2 ☐ I live alone
3 ☐ I live in a nursing home, hospital or other long term care home
4 ☐ Other

95. Do you have a long standing health condition? (Please include anything **other than your cancer** that has troubled you over a period of time or that could affect you over a period of time.)

1 ☐ Yes
2 ☐ No
3 ☐ Don't know/ can't say

96. Which, if any, of the following conditions do you have? **Tick all that apply.**

1 ☐ Alzheimer's disease or dementia
2 ☐ Angina
3 ☐ Arthritis
4 ☐ Asthma or other chronic chest problem
5 ☐ Blindness or visual impairment
6 ☐ Deafness or hearing impairment
7 ☐ Diabetes
8 ☐ Epilepsy
9 ☐ Heart condition
10 ☐ High blood pressure
11 ☐ Kidney disease
12 ☐ Learning difficulty
13 ☐ Liver disease
14 ☐ Long term back problems
15 ☐ Long standing mental health problem
16 ☐ Long standing neurological problem
17 ☐ Another long standing condition
18 ☐ I do not have any of these conditions

97. What was your employment status **before you were diagnosed with cancer**?

1 ☐ Full time employment
2 ☐ Part time employment
3 ☐ Homemaker
4 ☐ Student (in education)
5 ☐ Retired
6 ☐ Unemployed – and seeking work
7 ☐ Unemployed – unable to work for health reasons
8 ☐ Other

98. What is your employment status **currently**? (If on maternity or sick leave answer in relation to your **usual** employment status.)

1 ☐ Full time employment
2 ☐ Part time employment
3 ☐ Homemaker
4 ☐ Student (in education)
5 ☐ Retired
6 ☐ Unemployed – and seeking work
7 ☐ Unemployed – unable to work for health reasons
8 ☐ Other

99. If you are currently employed at the moment, are you:

1 ☐ Not working at all
2 ☐ Working less hours than usual
3 ☐ Working your usual hours
4 ☐ Working more hours than usual
5 ☐ This question does not apply to me

100. To which of these ethnic groups would you say you belong? **Tick ONE only.**

a. WHITE

1 ☐ British
2 ☐ Irish
3 ☐ Any other White background (Please write in box)

b. MIXED

4 ☐ White and Black Caribbean
5 ☐ White and Black African
6 ☐ White and Asian
7 ☐ Any other Mixed background (Please write in box)

c. ASIAN OR ASIAN BRITISH

8 ☐ Indian
9 ☐ Pakistani
10 ☐ Bangladeshi
11 ☐ Any other Asian background (Please write in box)

d. BLACK OR BLACK BRITISH

12 ☐ Caribbean
13 ☐ African
14 ☐ Any other Black background (Please write in box)

e. CHINESE OR OTHER ETHNIC GROUP

15 ☐ Chinese
16 ☐ Any other ethnic group (Please write in box)

This page has been left intentionally blank

COMMENTS

If you have anything else you would like to tell us about living with and beyond cancer, please do so here:

Please note: This section is **not** for general complaints; comments will **not** be actioned.

Questions 4 - 8 are EQ-5D-5L. UK (English) v.2 © 2009 EuroQol Group. EQ-5D™ is a trade mark of the EuroQol Group.

Questions 9 – 48 are taken from FACIT copyright 1987, 1997 by David Cella, PhD

Questions 50 – 70 are taken from the Social Difficulties Inventory. Originators of the Social Difficulties Inventory: Wright, E.P., Cull, A. and Selby, P
